# Psychiatric symptoms and comorbidities in patients with drug-resistant epilepsy in presurgical assessment—A prospective explorative single center study

**DOI:** 10.3389/fpsyt.2022.966721

**Published:** 2022-10-06

**Authors:** Fabian Friedrich, Ekaterina Pataraia, Susanne Aull-Watschinger, Sonja Zehetmayer, Lisbeth Weitensfelder, Clara Watschinger, Nilufar Mossaheb

**Affiliations:** ^1^Clinical Division of Social Psychiatry, Department of Psychiatry and Psychotherapy, Medical University of Vienna, Vienna, Austria; ^2^Department of Neurology, Medical University of Vienna, Vienna, Austria; ^3^Center for Medical Statistics, Informatics and Intelligent Systems, Medical University of Vienna, Vienna, Austria; ^4^Center for Public Health, Department of Environmental Health, Medical University of Vienna, Vienna, Austria

**Keywords:** psychopathology, epilepsy, neurosurgery, neuropsychiatry, psychiatric comorbidity, drug-resistant epilepsy

## Abstract

**Introduction:**

People with epilepsy (PWE) have a higher prevalence of psychiatric disorders. Some individuals with drug-resistant epilepsy might benefit from surgical interventions. The aim of this study was to perform an assessment of psychiatric comorbidities with a follow-up period of 12 months in patients with drug-resistant epilepsy, comparing those who underwent surgery to those who did not.

**Material and methods:**

We assessed psychiatric comorbidities at baseline, after 4 months and after 12 months. Psychiatric symptoms and diagnoses were assessed using SCID-Interview, Hamilton Rating Scale for Depression, Beck-Depression Inventory, Hamilton Anxiety Rating Scale, Prodromal-Questionnaire and the Global Assessment of Functioning Scale.

**Results:**

Twenty-five patients were included in the study, 12 underwent surgery, 11 were esteemed as being neurologically unqualified for surgery and two refused surgery. Patients in the no-surgery group were significantly older, reported more substance use, had significantly higher levels of anxiety and were more often diagnosed with a personality disorder. Age and levels of anxiety were significant predictors of being in the surgery or the no-surgery group. The described differences between surgery and no-surgery patients did not change significantly over the follow-up period.

**Discussion:**

These data point toward a higher expression of baseline psychiatric symptoms in drug-resistant PWE without surgery. Further studies are warranted to further elucidate these findings and to clarify potential psychotropic effects of epilepsy itself, drug-resistant epilepsy and of epilepsy surgery and their impact on psychopathology. Clinically, it seems highly relevant to include psychiatrists in an interdisciplinary state-of-the-art perioperative management of drug-resistant PWE.

## Introduction

Chronic somatic diseases are seen as one of the main causes of disability throughout the world (1). Several studies have reported a high prevalence of mental disorders in patients with chronic somatic illnesses (2). Gastrointestinal, metabolic, cardiovascular and pulmonary diseases, as well as musculoskeletal and neurological diseases go along with the highest prevalence rates of mental disorder [e.g. (3–7)]. Further, several studies show, that psychiatric comorbidity results in a poorer prognosis, increased resource utilization, higher costs, disability and poorer treatment compliance and treatment outcome (7–9).

Epilepsy is a chronic condition affecting nearly 70 million individuals worldwide with an incidence of around 50 per 100,000 per year in high income countries (9, 10). Further, people with epilepsy (PWE) have a significantly higher prevalence of comorbid somatic and psychiatric disorders including affective disorders, anxiety, and psychosis compared with the general population (9, 11–13). Depression and anxiety disorders are the most frequent comorbidities with life time prevalence rates between 30 and 35% in population-based studies (14, 15). Despite extensive pharmacological advances, current antiseizure medication are effective in just about 66% of patients in high-income countries (16, 17). Some individuals with drug-resistant epilepsy might benefit from surgical interventions (18). Progress within presurgical and neurosurgical technologies has evolved into significant improvements of effectivity of epilepsy surgery. The proportion of patients that are seizure-free after surgery ranges from 50 to 80% in selected groups (19). However, surgical treatment is still underused and potential candidates are often not referred to presurgical evaluation (9, 20, 21).

Not only do more than 50% of treatment-refractory PWE present with psychiatric symptoms, but psychiatric disturbances are also common after surgery, *de novo* or as exacerbation of pre-existing symptoms (11, 22, 23). The neurobiological mechanisms underlying the co-occurrence of epilepsy and psychiatric symptoms seem to be subjected to similar pathophysiological changes (9, 15). This has given rise to the concept of a possible bidirectional influence between psychiatric and epileptic symptoms (9, 12, 15). Furthermore, mental health conditions seem to affect significantly quality of life and social functioning in PWE (11). Ryvlin and Rheims (19) aimed at identifying predictors of epilepsy surgery outcomes and summarized that postoperative cognitive - and quality of life outcomes are influenced by antiseizure drugs and by psychiatric comorbidities. Nevertheless, the highest risk of postoperative psychiatric complications is often observed in patients with a preoperative psychiatric comorbidity (24, 25).

Data on psychiatric symptoms and syndromes in PWE with drug resistance in general, and pre-vs-post-operative data specifically, are generally sparse. Previous research mainly focused on psychiatric outcomes in patients who have undergone surgery, pointing toward a change in pattern and frequency of psychiatric symptoms after epilepsy surgery: While some data show up to $\frac{1}{4}$ th of patients with *de novo* psychiatric disorders post-operatively, others describe post-operative remission of 50% of pre-operative psychiatric symptoms (22, 24, 26).

The aim of this study was to perform a thorough und prospective assessment of psychiatric comorbidities with a follow-up period of 12 months in patients with drug-resistant epilepsy, comparing those who underwent surgery to those who did not.

## Materials and methods

### Design

In an open, prospective, explorative study, we investigated patients with drug-resistant epilepsy and possible indication for epilepsy surgery. We assessed psychiatric comorbidities at three time points: baseline (or pre-operative), after 4 months and after 12 months.

### Setting and participants

The study was carried out at the Department of Psychiatry and Psychotherapy in collaboration with the Department of Neurology, both Medical University of Vienna, Austria. The protocol was approved by the Local Ethics Committee (EK 1126/2013). All participants were thoroughly informed and signed the informed consent form thereafter.

The sample consisted of male and female participants aged 18–65 years with drug-resistant epilepsy, who were referred to a tertiary epilepsy center for presurgical epilepsy evaluation.

As part of standard care at the Epilepsy Monitoring Unit of the Department of Neurology patients undergo a presurgical assessment protocol including (a) clinical history and neurological examination, (b) neuropsychological assessment, (c) long-term video electroencephalography monitoring (VEEG), (d) structural magnetic resonance imaging (MRI), (e) functional MRI for assessment of language and memory functions, (f) fluorodeoxyglucose F18 positron emission tomography, and (g) computed perimetry. Each case was discussed in a multidisciplinary epilepsy board, consisting of neurologists, neuropsychologists, neurosurgeons, and neuroradiologists. After that the decision regarding the type of surgery was met.

We included PWE that were given recommendation for surgery and underwent surgery, PWE that were given recommendation for surgery and refused surgery and PWE who underwent thorough presurgical evaluation but because of discordant results of presurgical evaluation were not deemed as qualified for surgery.

### Clinical assessment and instruments

An experienced research assistant was in charge of the standardized clinical assessments and interviews. Additionally, all participants were seen by a psychiatrist at all time-points.

Current and previous psychiatric symptoms and diagnoses were assessed at baseline and at 12 months follow-up using the SCID Interview (Structured Clinical Interview for DSM IV Disorders, SCID I and II). The SCID interview is widely used, well validated and reliable (27, 28). SCID-I is used to determine major mental disorders including affective-, psychotic-, anxiety-, somatoform-, eating-, adjustment and substance abuse disorders as well as differential diagnoses. SCID-II interview is used to determine the occurrence of 12 different personality disorders.

The following instruments were used at all three time-points: In order to assess for potential clinical or subsyndromal depressive states and changes in depressive symptoms over time, the Hamilton Rating Scale for Depression [HAM-D (29)] and the self-rating Beck-Depression inventory [BDI (30)] were applied. These questionnaires are used to rate the severity of depression by probing symptoms like mood, feelings of guilt, suicide ideation, insomnia, agitation, weight loss or somatic symptoms. Anxiety-related symptoms like tensions, fears and cognitive functions were assessed using the Hamilton Anxiety Rating Scale [HAM-A (31)]. Since psychotic symptoms are more common in PWE than in the general population and subsyndromal psychotic symptoms can easily be overseen, the self-rating Prodromal-Questionnaire [PQ-16 (32)] was applied. This questionnaire was developed to detect help-seeking patients at clinically increased risk for psychosis. The PQ-16 includes nine questions regarding perceptual abnormalities, five questions on unusual thought content and delusional ideas and two on negative symptoms. Further, the Global Assessment of Functioning (GAF), a numeric scale included in DSM-IV to rate social, occupational and psychological functioning was used (33).

For HAM-D, BDI, HAM-A and PQ-16, higher scores represent a higher symptom load, whereas for the GAF a higher score represents higher psychosocial functioning.

### Procedures

Recruitment was done in a joint effort of the psychiatric and neurologic team of the respective departments, and in those cases that underwent surgery, also in accordance with the Department of Neurosurgery in order to plan preoperative baseline assessments. Patients were recruited consecutively between 2014 and 2016. All patients were assessed for baseline evaluation after recruitment during presurgical evaluation. Participants who underwent surgery were assessed 4 and 12 months after surgery, which was performed 2–4 months after completion of presurgical workup. Those who did not have surgery were assessed 4 and 12 months after their baseline assessment.

All patients who were deemed as having either a psychiatric disorder or being in clinical need of psychiatric help were offered such either directly at the Department of Psychiatry and Psychotherapy or at a local psychiatric care center, with a psychiatric specialist in practice or psychotherapist.

### Statistical analysis

The main analyses were done with patients grouped as observed over the study period, i.e., 12 patients who underwent surgery, vs. 13 who did not undergo surgery including the two patients who were recommended surgery but refused.

The primary question was to calculate the influence of the baseline scores of the clinical parameters HAM-D, BDI, HAM-A, PQ-16, and GAF, as well as age, on group affiliation to “surgery” vs. “no-surgery.” For each continuous variable two-sample *t*-tests were calculated. In addition, the influence of the binary variables SCID I, SCID II, and sex on group affiliation “surgery” vs. “no surgery” was investigated using Chi-square tests or Fisher exact tests. All continuous significant variables from the univariate analyses were further investigated and pairwise Pearson correlation coefficients were computed. In addition, a logistic regression analysis was performed to analyze the multiple influence of the significant variables (age and HAM-A) on “surgery yes/no” and odds ratios (OR) and 95% confidence intervals (CI) were reported.

To analyze potential changes in the continuous variables HAM-D, BDI, HAM-A, PQ-16, GAF between the two groups surgery yes/no over time during the follow-up period, a mixed model analysis with the fixed factors time (timepoints baseline, 4 months, 12 months), surgery group (yes/no) and the interaction of time and surgery group was applied. Time courses for each continuous variable plot the individual patient data and average values for the patient groups. The patient was considered as random factor. Second, an analysis of covariance was performed with the difference of each continuous variable (between baseline and 12-months value) as target variable and the independent variables group and the corresponding baseline value.

Changes over time in the psychiatric diagnoses assessed with the SCID I were performed using the baseline and 12-months value. Potential differences between groups were calculated using Fisher exact text. Since the number of changes over time in personality disorders assessed with the SCID II was too small, no further analyses were performed.

In accordance with current guidelines and recommendations regarding changes in anti-seizure medication in PWE with drug-resistance, medication was not changed during the whole study period in any of the participants and was therefore not included in the statistical analysis (34).

The significance level was set to 0.05 for all analyses. Due to the exploratory nature of the study, no adjustment for multiple testing was performed. For the analysis the R program was used (https://cran.r-project.org).

## Results

At total of 25 patients were included in the study. Four patients did not complete the study (two dropped out between baseline and month 4 and two between month 4 and month 12). Of the 25 patients, 12 underwent surgery, whereas 13 did not (11 of which were esteemed as being neurologically unqualified for surgery and two of which refused surgery).

### Baseline results

Demographic and clinical information are presented in Tables 1A,B.

**Table 1A:** Sociodemographic data at baseline.

	**No surgery**	**Surgery**	***p*-value**
*n*	13	12	
Age [mean (SD)]	46.92 (12.96)	34.92 (11.48)	0.007^*^
Sex (% male)	61.5	33.3	0.238
Completed secondary education incl apprenticeship %	84.7	100	0.61
In a partnership %	53.9	66.6	0.45
Having children %	53.8	41.7	0.695
Living alone %	23.1	16.7	0.325
Work situation: unemployed or early retirement %	38.5	25	0.41
Suicide attempts	0	0	n/a
Substance use incl nicotine %	92.3	50	0.03^*^
Family history of psych. dis.	23.1	17.7	1
Seizure frequency >1/week	76.9	83.3	1
Generalized seizures	53.8	75	0.411

* Significant value, n/a means no data available.

**Table 1B:** Clinical data at baseline.

**Patient #**		**Sex**	**Diagnosis**	**Seizure frequency at BL**	**ASM**
# 1	Surgery group	f	BPVH, lesionectomy right temporo-occipital after invasive monitoring, Histology: FCD	1–3/day	LVT, LCS
# 2	Surgery group	f	MTS left, sAHE, Histology: HA Type 1 ILAE	1–6/week	LVT, LCS, CBZ
# 3	Surgery group	m	Focal MRI-negative TLE right, AMT, Histology: no pathology	4–11/year	LVT, CBZ
# 4	Surgery group	f	Focal MRI-negative TLE right, AMT, Histology: unspecific changes	1–6/week	LTG
# 5	Surgery group	f	MTS left, sAHE, Histology: HA Type 1 ILAE	1–6/week	LVT, LCS, ZNS
# 6	Surgery group	f	TLE with bilateral seizure onset and MTS left, AMT left, Histology FCD Typ 1	1–6/week	LVT, OXC
# 7	Non-surgery group	f	Focal MRI-negative epilepsy with right hemispheric onset, seizure onset unclear after invasive monitoring	1–6/week	LVT, LCS, ESL
# 8	Non-surgery group	m	Focal MRI-negative epilepsy with left posterior temporal seizure onset	1–3/month	VPA, PER
# 9	Non-surgery group	m	TLE right, AMT 5 years before, bitemporal seizure onset	1–3/month	LVT, PER
# 10	Non-surgery group	f	Focal MRI-negative FLE left	1–6/week	LVT, LCS
# 11	Surgery group	m	Focal MRI-negative TLE left, sAHE, Histology: unspecific changes.	1–6/week	LCS, OXC
# 12	Non-surgery group	f	Focal MRI-negative bitemporale epilpesy	1–6/week	LVT, OXC
# 13	Surgery group	f	Focal MRI-negative TLE right, AMT after invasive monitoring, Histology: FCD	1–6/week	GBP, OXC
# 14	Non-surgery group	m	Focal MRI-negative FLE	1–3/year	LVT, OXC
# 15	Non-surgery group	m	Focal MRI-negative epilepsy	1–6/week	LVT, LCS
# 16	Non-surgery group	m	Focal MRI-negative FLE left	1–3/day	LCS
# 17	Surgery group	f	MTS left, sAHE, Histology: HA Type 1 ILAE	4–11/year	LVT, LCS
# 18	surgery group	f	Lesional TLE right, lesionectomy, Histology: ganglioglioma	1–3/month	LTG
# 19	Non-surgery group	m	BPVH with right occipital seizure onset	1–6/week	LVT, LCS
# 20	Non-surgery group	m	Focal MRI-negative bilateral TLE	1–3/month	LVT, LCS
# 21	Non-surgery group	f	Focal MRI-negative FLE right	1–6/week	LVT, CBZ
# 22	Non-surgery group	m	Focal MRI-negative bilateral TLE	1–6/week	LVT, LCS, PER
# 23	Non-surgery group	f	Malformation of cortical development left hemispheric and right temporal	1–6/week	LVT, LTG, PER
# 24	Surgery group	m	MTS right, sAHE, Histology: HA Type 1 ILAE	1–6/week	LVT, PER
# 25	Non-surgery group	m	Focal MRI-negative TLE left and psychogenic non-epileptic seizures	1–3/day	LTG

MTS, mesial temporal sclerosis; TLE, temporal lobe epilepsy; sAHE, selective amygdalohippocampectomy; AMT, anteromesial temporal lobe resection; BPVH, bilateral periventricular heterotopia; FCD, focal cortical dysplasia; FLE, frontal lobe epilepsy; ASM, antiseizure medication; LVT, levetiracetam; LCS, lacosamide; CBZ, carbamazepine; ESL, Eslicarbacepine; GBP, gabapentin; OXC, oxcarbazepine; ZNS, zonisamid; PER, perampanel; LTG, lamotrigine.

There were no differences in sex distribution or in the assessed sociodemographic variables between groups, with the exception of age being significantly higher (*p* = 0.007) and substance use including nicotine being more frequent in patients in the no-surgery group (*p* = 0.030). Regarding clinical parameters, on a descriptive level, PWE in the no-surgery group show higher baseline scores on depression and anxiety scales (HAM-D, HAM-A and BDI), as well as lower psychosocial functioning represented by lower scores on the GAF. However, in the univariate analysis, only baseline HAM-A scores (*p* = 0.015) were significantly different between the surgery vs. no-surgery group, with individuals in the no-surgery group having higher anxiety levels. A significant difference was also observed in the frequency of personality disorder diagnoses (SCID II) with none in the surgery group vs. 36.4% in the no-surgery group (*p* = 0.026). There were no differences in frequency of past or current psychiatric disorders assessed by the SCID I (see also Table 2). In neither of the groups were there any cases of present or past hypomania, mania, dysthymia, psychotic episodes, OCD, PTSD, generalized anxiety disorder, somatoform disorder, somatization disorder, pain disorder, body dysmorphophobia, eating disorder. The most common SCID I diagnoses were depressive disorders, anxiety disorders, alcohol abuse, adaptation disorder, with single to maximum three cases each.

**Table 2:** Clinical variables over time.

	**No surgery**	**Surgery**	**No surgery**	**Surgery**	**No surgery**	**Surgery**
	**Baseline**	**Baseline**	**Month 4**	**Month 4**	**Month 12**	**Month 12**
HAMD [mean (SD)]	9.69 (6.61)	5.92 (4.06)	8.55 (5)	4.33 (4.39)	10.45 (4.06)	7.56 (4.79)
BDI [mean (SD)]	7.62 (5.24)	5.33 (4.56)	5.09 (3.53)	2.92 (3.39)	7.36 (5.71)	3.7 (3.97)
HAMA [mean (SD)]	12.08 (4.72)	7.08 (4.17)	10.73 (5.44)	5.25 (3.38)	10.91 (4.81)	6.6 (4.43)
PQ16 [mean (SD)]	3.23 (2.35)	1.56 (1.24)	1.73 (1.42)	2.56 (3.47)	2.10 (1.85)	1.44 (2.07)
GAF [mean (SD)]	65.23 (13.18)	75.17 (14.24)	65.18 (15.63)	72.25 (12.78)	65.45 (15.36)	67.4 (19.55)
SCID I diagnosis %	7 (53.8)	6 (50.0)			8 (72.7)	8 (80)
SCID II diagnosis %	4 (30.8)	0 (0.0)			2 (18.2)	1 (10%)

Correlations between the influential variables HAM-A and PQ-16 scores were higher in the no-surgery group (0.83 vs. 0.37 in the surgery group). The negative correlation between age and PQ-16 showed a moderate level in both groups (−0.31). Age and HAM-A also correlated moderately in the surgery group (−0.34). In the logistic regression analysis including age and HAM-A both variables have a significant influence on group allocation to surgery vs. no-surgery (age: *z*-value 2.010; *p*-value 0.044; OR 1.105; 2.5% CI 1.002; 97.5% CI 1.219/HAMA-score; *z*-value 2.137; *p*-value 0.033; OR 1.361; 2.5% CI 1.026; 97.5 % CI 1.804).

In order to account for the status of neurological operability of patients at baseline a further analysis was performed grouping the patients according to their recommendation at baseline, i.e. 14 operable patients and 11 that were not recommended for surgery – regardless of whether they ended having surgery or not. There were no relevant differences in our findings in this further analysis.

### Follow-up analysis

**Figure 1:**
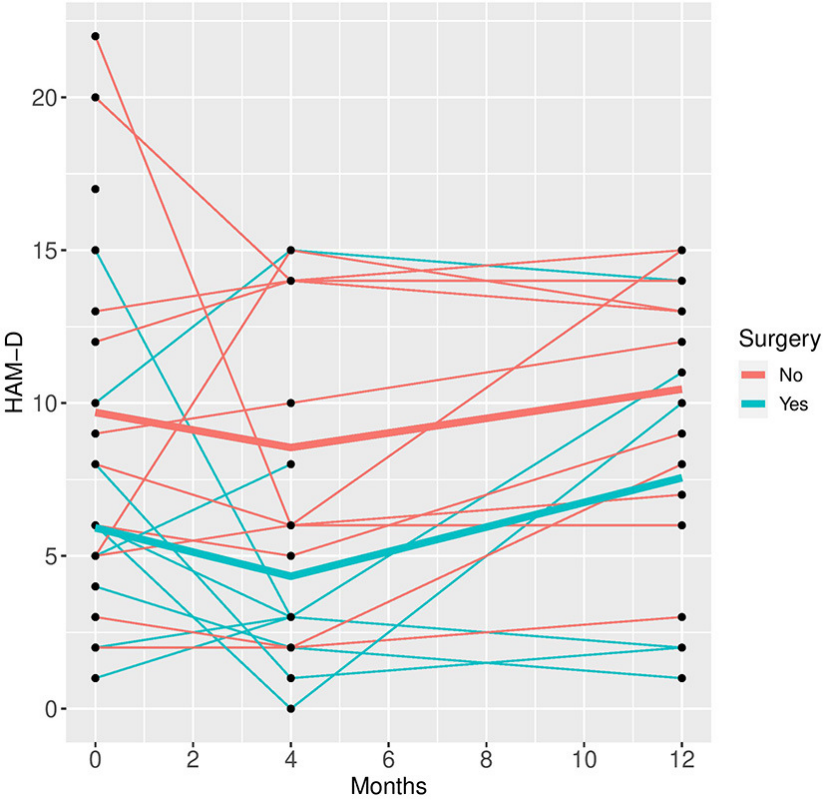
HAM-D time course for individual patient data (thin lines) and averaged values (bold lines).

**Figure 2:**
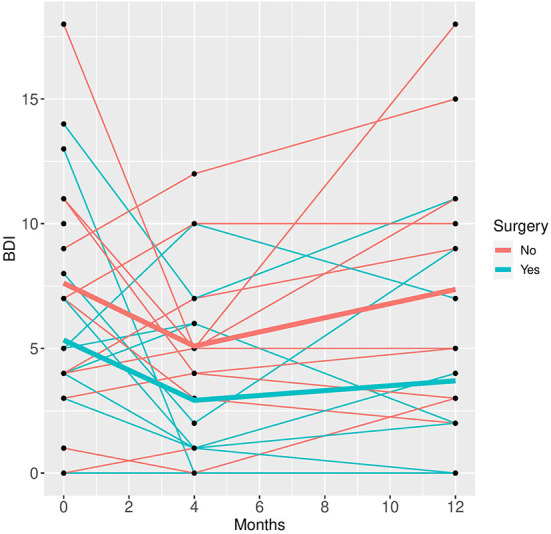
BDI time course for individual patient data (thin lines) and averaged values (bold lines).

**Figure 3:**
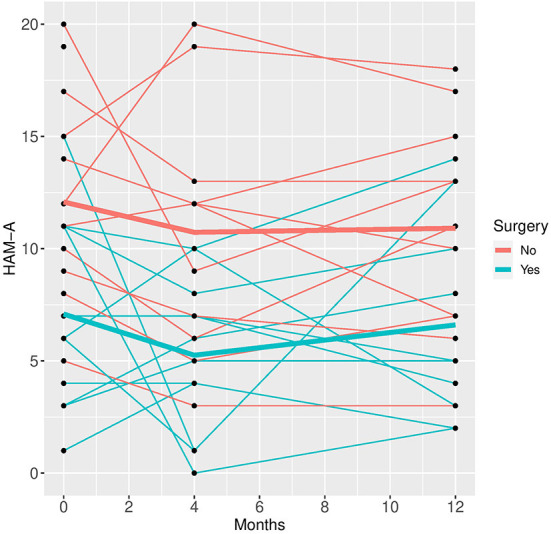
HAM-A time course for individual patient data (thin lines) and averaged values (bold lines).

**Figure 4:**
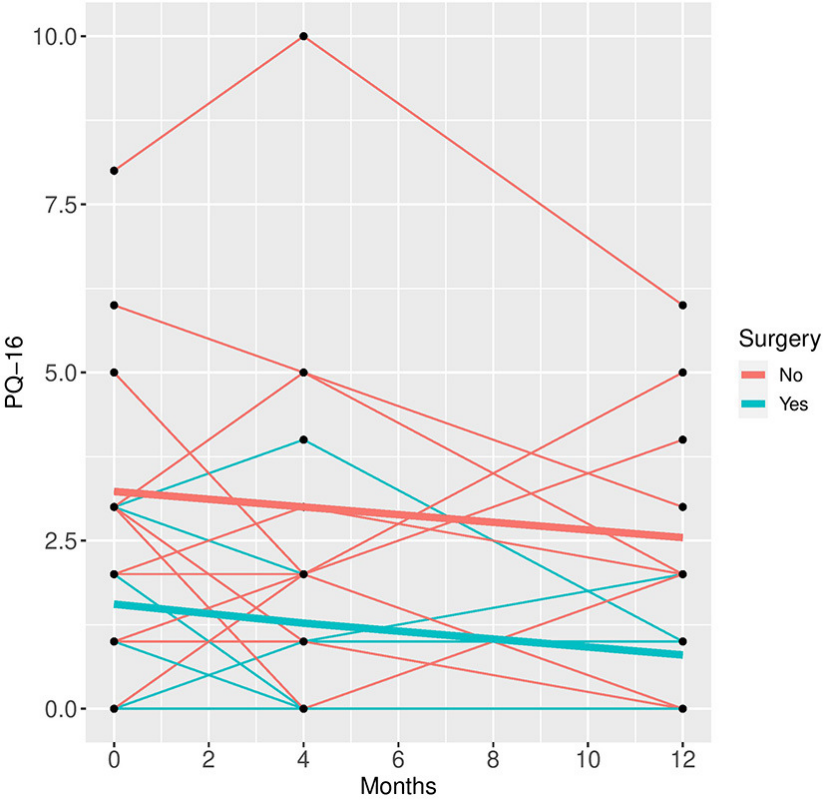
PQ-16 time course for individual patient data (thin lines) and averaged values (bold lines).

**Figure 5:**
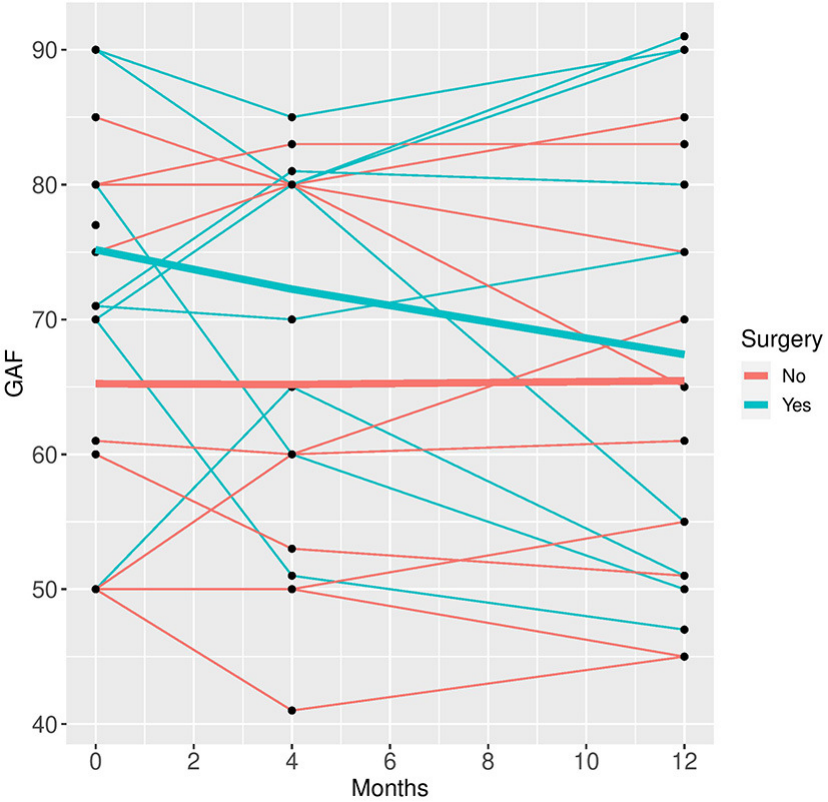
GAF time course for individual patient data (thin lines) and averaged values (bold lines).

In the mixed model analysis using the fixed factors time, surgery and the interaction of time and surgery group the interaction was not significant in any of the models, implying that the differences between groups were not changing significantly over time. Therefore, mixed models were repeated with the fixed factors time and group but without the interaction factor (see Figures 1–5).

See Table 2 for scores in clinical variables over the follow-up period.

Patients who did not undergo surgery have on average higher HAM-D values with no significant changes over both groups over time. The analysis of covariance is not significant for both analysis sets (*p* = 0.57, or 0.85, respectively), implying that the change of HAM-D from time 0 to time 12 is not significantly different between the surgery groups. The same results apply for HAM-A and PQ-16. Note that the analysis of covariance is not significant for any of the continuous variables. Only the mixed model analysis of BDI scores shows a significant difference between baseline and 4 months values (*p* = 0.019), no other significant influence of time can be found. The analysis of the change of the SCID I values shows no significant result (see Table 3).

**Table 3:** Mixed model analysis.

**Mixed models**	**Estimate**	***t*-Value**	***p*-Value**
**HAM-D**			
Surgery yes/no	−3.6833	−2.201	0.0384*
Timepoint 4 months	−1.3385	−1.368	0.1787
Timepoint 12 months	1.2619	1.228	0.2260
**BDI**
Surgery yes/no	−2.9174	−1.959	0.0625
Timepoint 4 months	−2.2647	−2.447	0.0185*
Timepoint 12 months	−0.7417	−0.777	0.4413
**HAM-A**			
Surgery yes/no	−4.9821	−3.353	0.00289**
Timepoint 4 months	−1.5846	−1.668	0.10274
Timepoint 12 months	−0.8406	−0.858	0.39569
**PQ-16**			
Surgery yes/no	−1.7635	−2.473	0.0216*
Timepoint 4 months	−0.1834	−0.480	0.6339
Timepoint 12 months	−0.6053	−1.558	0.1269
**GAF**
Surgery yes/no	7.071	1.286	0.211
Timepoint 4 months	−1.303	−0.584	0.562
Timepoint 12 months	−2.386	−1.034	0.307
**Analysis of covariance**			
**HAM-D**			
Surgery yes/no	−1.0288	−0.571	0.57548
HAM-D baseline	−0.5313	−3.218	0.00504**
Adjusted *R*-squared			0.3161
**BDI**
Surgery yes/no	−2.4687	−1.392	0.1808
BDI baseline	−0.3428	−1.778	0.0923
Adjusted *R*-squared			0.1027
**HAM-A**			
Surgery yes/no	−0.7368	−0.350	0.731
HAM-A baseline	−0.3385	−1.491	0.153
Adjusted *R*-squared			0.0305
**PQ-16**			
Surgery yes/no	−1.0708	−1.357	0.19361
PQ-16 baseline	−0.5777	−3.051	0.00763**
Adjusted *R*-squared			0.2891
**GAF**			
Surgery yes/no	−4.0333	−0.633	0.535
GAF baseline	−0.2095	−0.899	0.380
Adjusted *R*-squared			−0.01781

HAM_D baseline, BDI_baseline, HAM-A_baseline, PQ-16_baseline and GAF_baseline refer to the influence of the baseline scores of each of these variables on the change of the scores over time (i.e., change between baseline and 12-months follow-up).

^*^p < 0.05,

^**^p < 0.01.

## Discussion

The aim of this study was to assess the presence of psychiatric comorbidities over a follow-up period of 1 year in patients with drug-resistant epilepsy comparing those who did and those who did not undergo epilepsy surgery.

Baseline results of our study show that, indeed, drug-resistant PWE that underwent surgery and those that did not, differed. PWE in the “no surgery” group were significantly older, reported more substance use, had significantly higher levels of anxiety and were more often diagnosed with a personality disorder. Age and levels of anxiety were significant predictors whether someone was in the surgery or the no-surgery group.

Our data point toward a higher expression of psychiatric symptoms, such as anxiety, substance use and personality disorders, in PWE without surgery. Since most patients in the no-surgery group in this study were those for whom epilepsy surgery was not recommended after thorough evaluation, one might assume that the differences in psychopathology might derive from the disappointment of refusal of surgery, from a sense of defeatism. However, at baseline, the study participants were still being evaluated and mostly had not received the results of their examinations regarding possible surgery yet.

Our baseline findings also imply the necessity and the importance of close psychiatric monitoring in PWE with no surgery options, as they seem to express more psychiatric symptoms than other patients do.

The question that arises is whether these patients express more psychiatric symptoms because of their chronic illness or whether their symptoms are organically part of their index illness. The pathophysiological mechanisms of seizure activity are not fully understood, epilepsies remain a diverse set of disorders (9). The bidirectional hypothesis suggests, that psychiatric disorders are not simply caused by reactions to epilepsy and its consequences, but might, in some patients, be related to the same underlying pathophysiology that is involved in epileptic seizures and in the expression of psychiatric disorders (15, 35). With respect to our sample (40% of our patients had a TLE, *n* = 10), it remains unclear, whether higher expression of psychiatric symptoms may be interpreted as an associated dysregulation of epilepsy itself – which might or might not be more pronounced in our no-surgery group. Indeed, common networks involved in the expression and regulation of mood are affected in Temporal Lobe Epilepsy (TLE). However, the causality of the findings were not assessed in this study and were not the aim of this study (15).

Generally, it often remains challenging to distinguish between episodic, ictal phenomena and an ongoing underlying psychiatric affective or anxiety disorder. This may confound assessments of psychiatric comorbidities, especially with a cross-sectional observation when psychiatric and neurological symptoms and their treatment might fluctuate (35). Despite the widely recognition of high prevalence rates of common psychiatric disorders in PWE, they remain highly underdiagnosed and untreated (11, 14). On the one hand, findings suggest a limited training of neurologists and psychiatrists on psychiatric aspects of neurologic disorders and neurologic aspects of psychiatric disorders (14). On the other hand, the inclusion of psychiatrists in diagnosis and treatment of PWE seems essential to improve interdisciplinary approach and outcome. Psychiatric evaluation differs significantly from neuropsychological screening approach. Patients' life time psychiatric history and family history, concomitant personality disorders and definition of categorical psychiatric disorder is rarely established in neuropsychological settings (36). As a matter of fact, research on psychiatric symptoms in PWE are most often done without psychiatric experts and with self-ratings of more general psychological scales. Thus, one of the strengths of this study is the use of standardized and well-validated psychiatric rating scales, assessments by trained researchers and a psychiatric evaluation at each timepoint (36).

Another question that arises is whether pre-existing psychiatric disorders might to some extent influence neurologists' and neurosurgeons' decision on the operability of PWE. Higher levels of anxiety and personality disorders may be defined as “soft markers” of surgery success. The presence of an active and severe psychiatric condition is considered as a relative contraindication to epilepsy surgery. Nevertheless, surgery can be recommended, if the disorder is resolved (21). This underlines the critical necessity of psychiatrists being included in the perioperative management of PWE (36).

In our study, baseline assessments were done during presurgical evaluation, hence decisions on operability were not conclusive yet and will not have influenced our baseline findings.

A further strength of our study is the longitudinal aspect of follow-up assessments over a period of 12 months, allowing for a more in-depth look at how psychiatric symptoms might or might not change and develop over time in those who had surgery vs. those who didn't. Interestingly, time had hardly any influence on the differences observed at baseline. The latter were only somewhat emphasized over time. Psychiatric outcome following surgery has been discussed controversially. While some authors reported deterioration or no change (37), others have found improvements. Ramos-Perdigues et al. (25) described improvements in anxiety and depression scores 12 months after epilepsy surgery in seizure-free patients, albeit a lack of changes in major psychiatric diagnoses (assessed with the SCID I). Contrary to our sample, their sample did not differ with respect to age or psychiatric symptoms assessed at baseline. While Ramos-Perdigues' sample size was larger (*n* = 152), the differences in study settings might explain some of the strengths and challenges of our study: Our patients were assessed by a psychologist well trained in psychiatric rating scales. Also, not only did we examine the presence of SCID I-diagnoses and specific psychiatric symptoms, but also the occurrence of personality disorders. Furthermore, our study was conducted within a psychiatric department with all the benefits and challenges that entails.

Alternatively, surgery may play its own role in influencing the regulation of emotions. In a state of excessive activity in chronic seizures inhibitory activity of serotonin and noradrenaline is observed. The decline of those neurotransmitters has been shown to facilitate the kindling process of seizure foci. Forced normalization is the most commonly attributed mechanism of mood disturbances. Surgery seems to eradicate the mood stabilizing effect of seizures through seizure freedom (21, 24, 38). The few studies on longitudinal psychiatric symptoms after epilepsy surgery tend to report a certain amount of postoperative *de novo* psychiatric symptoms occurring after 3–6 months. Interestingly, the only significant change over time in our study was a drop in self-reported depressive symptoms (BDI) in both groups. Our data confirm Bujarski et al.'s (37) findings regarding a lack of significant improvement in psychiatric conditions after surgery. Antiseizure medication remained unchanged over the follow-up period, as per current guidelines, therefore, potential effects of these can be regarded as not relevant for our results. Since there do not seem to be any relevant changes in psychiatric symptoms over the observed time in either of our groups, special attention should be given to maintain longer term psychiatric observation especially in the no-surgery PWE.

As factors limiting interpretation of our findings the small sample size needs mentioning. Indeed, albeit the joint recruitment efforts of all involved, the sample size remained small, pointing toward the difficulties in performing larger studies in severely and chronically ill patients. Reasons for refusal of participation were most commonly reluctance to be assessed in a psychiatric department. However, these reasons were not assessed systematically. Further difficulties in recruitment concerned changes in neurosurgery schedules on a short-term, making preoperative assessments not always possible. Also, only patients with focal epilepsy were included, limiting a generalizability of our findings to PWE with other forms of epilepsy. Despite the fact that literature considers a postoperative adaptation phase to end after 12 months, a longer follow-up period would provide further details into long-term psychiatric symptoms and sequelae and might present our findings in a different perspective.

Further studies seem necessary to clarify the potential psychotropic effects of epilepsy itself and of epilepsy surgery and to broaden the knowledge on the impact of drug-resistant epilepsy on psychiatric symptoms. From a clinical perspective it seems necessary and relevant to include psychiatrists in an interdisciplinary state-of-the-art perioperative management of drug-resistant PWE.

## Data availability statement

The original contributions presented in the study are included in the article/supplementary material, further inquiries can be directed to the corresponding author.

## Ethics statement

The studies involving human participants were reviewed and approved by Ethikkommission der Medizinischen Universität Wien. The patients/participants provided their written informed consent to participate in this study.

## Author contributions

Concept and design: FF, EP, SA-W, and NM. Acquisition, analysis, interpretation of data, and drafting of the manuscript: FF, EP, SZ, SA-W, LW, CW, and NM. Statistical analysis: SZ. Obtained funding: NM. All authors contributed to the article and approved the submitted version.

## Funding

This research was supported by a grant of the Medizinisch-Wissenschaftlicher Fonds des Bürgermeisters der Bundeshauptstadt Wien (Grant number 13076).

## Conflict of interest

The authors declare that the research was conducted in the absence of any commercial or financial relationships that could be construed as a potential conflict of interest.

## Publisher's note

All claims expressed in this article are solely those of the authors and do not necessarily represent those of their affiliated organizations, or those of the publisher, the editors and the reviewers. Any product that may be evaluated in this article, or claim that may be made by its manufacturer, is not guaranteed or endorsed by the publisher.
